# Special Issue “The Role of Natural Products in Drug Discovery”

**DOI:** 10.3390/ijms27010246

**Published:** 2025-12-25

**Authors:** Luciana Scotti, Marcus Tullius Scotti

**Affiliations:** Cheminformatics Laboratory, Postgraduate Program in Natural Products and Synthetic Bioactive Compounds, Federal University of Paraíba, Campus I, João Pessoa 58051-970, PB, Brazil

## 1. Background

Natural products—bioactive compounds derived from plants, microbes, fungi, and marine organisms—have shaped the pharmaceutical landscape for decades. Many of the world’s most important drugs, including antibiotics, anticancer agents, and immunosuppressants, originate from natural sources. Their evolutionary refinement endows them with unique chemical features that synthetic libraries often cannot fully replicate. Classic examples span multiple therapeutic areas: antibiotics such as penicillin and tetracyclines arise from microbial metabolites; anticancer agents like paclitaxel (from yew trees) and doxorubicin (from *Streptomyces bacteria*) also trace their origins to nature; and numerous antiparasitic drugs follow this pattern. Even today, a substantial proportion of FDA-approved drugs are natural or natural-product-inspired, underscoring their enduring relevance [1,2,3,4,5,6,7].

Natural compounds inhabit a broader and more complex chemical space than typical synthetic molecules. They offer several key advantages, including high structural diversity with intricate ring systems and rich stereochemistry; increased three-dimensionality that enhances receptor binding; and novel molecular scaffolds that inspire entirely new drug classes. This diversity makes natural products particularly valuable as lead compounds—robust starting points for medicinal chemistry optimization [1,2,3,4,5,6,7].

The integration of natural products with modern technologies represents a dynamic convergence of tradition and innovation. Age-old biological resources are increasingly enhanced through advances in biotechnology, nanotechnology, artificial intelligence, and other emerging fields. State-of-the-art extraction and purification methods enable scientists to isolate bioactive compounds with unprecedented precision, while nanotechnology supports the design of targeted drug-delivery systems built from natural molecules. Artificial intelligence accelerates discovery by analyzing vast datasets to predict the therapeutic potential of natural substances, thereby reducing both research time and cost. Beyond healthcare, natural products are being incorporated into sustainable materials, renewable energy solutions, and eco-friendly packaging, driven by innovations in green chemistry and engineering [1,2,3,4,5,6,7].

This synergy not only preserves the wisdom embedded in traditional practices but also ensures scalability, efficiency, and sustainability in addressing global challenges. Ultimately, the fusion of natural products with modern technologies illustrates how science can harmonize with nature to generate solutions that are both cutting-edge and environmentally responsible.

## 2. Highlights of Research Contributions

Drs Sun and Gokhale explored the molecular targets of flavonoids to gain insight into the mechanism of action behind their biological effects (contribution 1). In this study, a novel class of resorcinol-based flavonoid compounds was identified as a potent inhibitor of human DNA ligase activity. Human DNA ligases are crucial in the maintenance of genetic integrity and cell fate determination.

Drs Estévez and co-workers explored the photoprotective properties of two different *Aspalathus linearis* extracts (fermented and unfermented) individually, and then in combination, in a simplified model assessing Normal Human Dermal Fibroblast survival after UVB radiation (contribution 2).

We reported a work that assessed the antinociceptive and anti-inflammatory effects of 4-hydroxycoumarin in animal models at doses of 25, 50, and 75 mg/kg. 4-HC significantly reduced abdominal contortions induced by acetic acid and decreased nociceptive rubbing in orofacial pain models induced by formalin, glutamate, and capsaicin (contribution 3). 

Contribution 4 shows a study by Drs Ding et al. The authors investigated the active components of *Crocus alatavicus* and potential targets through a combination of network pharmacology, molecular docking technology combined with molecular dynamics simulation, and binding free energy analyses. A total of 253 active ingredients from *C. alatavicus* were screened, and 1360 associated targets were predicted through systematic searches conducted using TCMSP, SwissDrugDesign, and SymMap, which were integrated to construct a pharmacological network to dissect the relationships among active components, targets, diseases, and pathways; we found prostate cancer-related genes were significantly enriched among the targets.

Drs Jung-Wook Kang and In-Chul Lee investigated the ability of Pentagalloylglucose (PGG) to selectively inhibit hyperpigmentation through the regulation of melanogenesis in melanocytes. PGG inhibited melanin production in α-melanocyte-stimulating hormone (α-MSH)-induced B16F10 melanocyte-stimulating cells (contribution 5).

The lemon flavonoid extract Eriomin^®^ (LE), which is rich in eriocitrin, has demonstrated antioxidant and anti-inflammatory properties in both animal and human studies. Given the established interplay among aging, oxidative stress, and inflammation, Dr Trifunovic et al. investigated the influences of LE on the pituitary–adrenal (PA) axis in aged rats and its potential to mitigate age-related physiological changes in this system. The effects of LE (40 mg/kg/day suspended in sunflower oil) on the morphofunctional properties of the PA axis were studied in 24-month-old male Wistar rats following four weeks of oral treatment (contribution 6).

The review by Drs Mesta-Corral et al. was contribution 7. The work provides a review of the history of the wine industry and its current activities, describes the wine production process, and outlines the waste generated during this process. The fermentation process is described as a biotechnological alternative for the valorization of these residues.

In contribution 8, Dr Efimova and co-workers elucidated the effects of natural protoberberine alkaloids (rotundine, berberine, and nitidine) and cinnamic acid derivatives (ethyl-4-methoxycinnamate and osthole) found in Vietnamese medicinal plants, on the boundary potential of lipid bilayers and phase behavior of membrane lipids. 

We, the Guest Editors, gratefully acknowledge all contributing authors for their high-quality submissions, the expert reviewers for their insightful evaluations, and the IJMS editorial team for their professional support throughout the publication process.
